# Unveiling the Morphology of Carbon-Supported Ru Nanoparticles by Multiscale Modeling

**DOI:** 10.1021/acs.nanolett.3c03796

**Published:** 2024-01-29

**Authors:** Wenye Xuan, Yu-Hao Liu, Shih-Yuan Chen, Matthew S. Dyer, Hsin-Yi Tiffany Chen

**Affiliations:** † Department of Engineering and System Science, National Tsing Hua University, Hsinchu 30013, Taiwan; ‡ School of Chemistry, University of Liverpool, Crown Street, Liverpool L69 7ZD, United Kingdom; § Energy Catalyst Technology Group, Energy Process Research Institute (EPRI), National Institute of Advanced Industrial Science and Technology (AIST), 16-1 Onogawa, Tsukuba, Ibaraki 305-8569, Japan; ∥ Materials Innovation Factory, University of Liverpool, 51 Oxford Street, Liverpool L7 3NY, United Kingdom; ⊥ College of Semiconductor Research, National Tsing Hua University, Hsinchu 30013, Taiwan; # Department of Materials Science and Engineering, National Tsing Hua University, Hsinchu 30013, Taiwan

**Keywords:** metal nanoparticles, DFT calculation, global minimal search, AIMD, DPMD, multiscale modeling

## Abstract

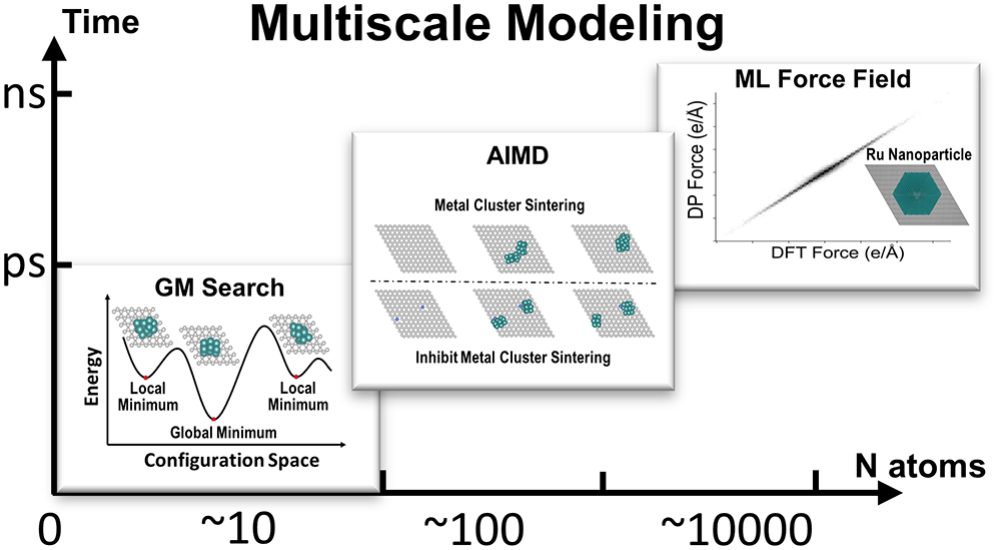

Simulating the behavior of metal nanoparticles on supports is crucial for boosting their catalytic performance and various nanotechnology applications; however, such simulations are limited by the conflicts between accuracy and efficiency. Herein, we introduce a multiscale modeling strategy to unveil the morphology of Ru supported on pristine and N-doped graphene. Our multiscale modeling started with the electronic structures of a supported Ru single atom, revealing the strong metal–support interaction around pyridinic nitrogen sites. To determine the stable configurations of Ru_2–13_ clusters on three different graphene supports, global energy minimum searches were performed. The sintering of the global minimum Ru_13_ clusters on supports was further simulated by ab initio molecular dynamics (AIMD). The AIMD data set was then collected for deep potential molecular dynamics to study the melting of Ru nanoparticles. This study presents comprehensive descriptions of carbon-supported Ru and develops modeling approaches that bridge different scales and can be applied to various supported nanoparticle systems.

Advances in nanotechnology have significantly improved the control of nanoparticle morphology, enabling the manipulation of the crystal structures and facets. Metal nanoparticles can be impregnated onto supports through metal–support interactions (MSI), resulting in supported metal catalysts that are widely applied in industrial refining and fine chemical synthesis processes. In the past few decades, carbon-supported Ru catalysts that contain 2–3 nm Ru nanoparticles with abundant B5 sites for N_2_ activation and dissociation have exhibited higher activity in commercial Haber–Bosch ammonia synthesis under milder conditions compared to that of conventional iron catalysts.^1−3^ Recent studies combining experimental methods and theoretical calculations have provided further insights into supported catalysts of a Ru single metal atom (SMA) and atomic clusters, revealing their unique MSI that influence structural and electronic properties and, consequently, exhibit distinct gas sorption and catalytic behaviors. Li et al. demonstrated that a Ru_3_ cluster catalyst might perform ammonia synthesis through the associative mechanism.^4^ Huang et al. showed that the mechanism of ammonia decomposition over MgO-supported Ru catalysts depends on the size and morphology of Ru.^5^

Hence, understanding and controlling the structure of supported Ru catalysts are crucial for the study and enhancement of their catalytic performance. Among a variety of support materials, nitrogen-doped graphene has recently garnered significant attention owing to its capability in achieving atomically dispersed metal catalysts.^4,6−8^ Substantial efforts have been focused on precisely characterizing pristine and N-doped graphene-supported metal structures using both experimental and theoretical approaches.^9−12^ However, the conflicts between accuracy and efficiency of simulation have substantially limited the precision and scope of modeling in previous theoretical studies. More specifically, the computationally intensive self-consistent field calculations in density functional theory (DFT) restrict the scale of first-principles calculations to a few hundred atoms. Conversely, molecular dynamics with classical force fields can simulate systems containing millions of atoms but at the expense of reduced accuracy, particularly for those models discussing surface or interface phenomena.

In this work, we present a multiscale modeling approach that strikes a balance between accuracy and efficiency at each scale, enabling us to explore the morphology of Ru supported on pristine and N-doped graphene. As illustrated in Figure 1, the multiscale modeling started with DFT optimization of supported Ru SMAs and their electronic structure analysis. Subsequently, we transitioned to a global minimum (GM) search for supported Ru atomic clusters. The resulting GM atomic cluster models were then employed in ab initio molecular dynamics (AIMD) simulations to investigate the particle coalescence process. The data gained from first-principles calculations in earlier steps were harnessed to construct a machine learning-based force field for deep potential molecular dynamics (DPMD), enabling the determination of the thermal stability of nanoscale Ru particles.

**Figure 1:**
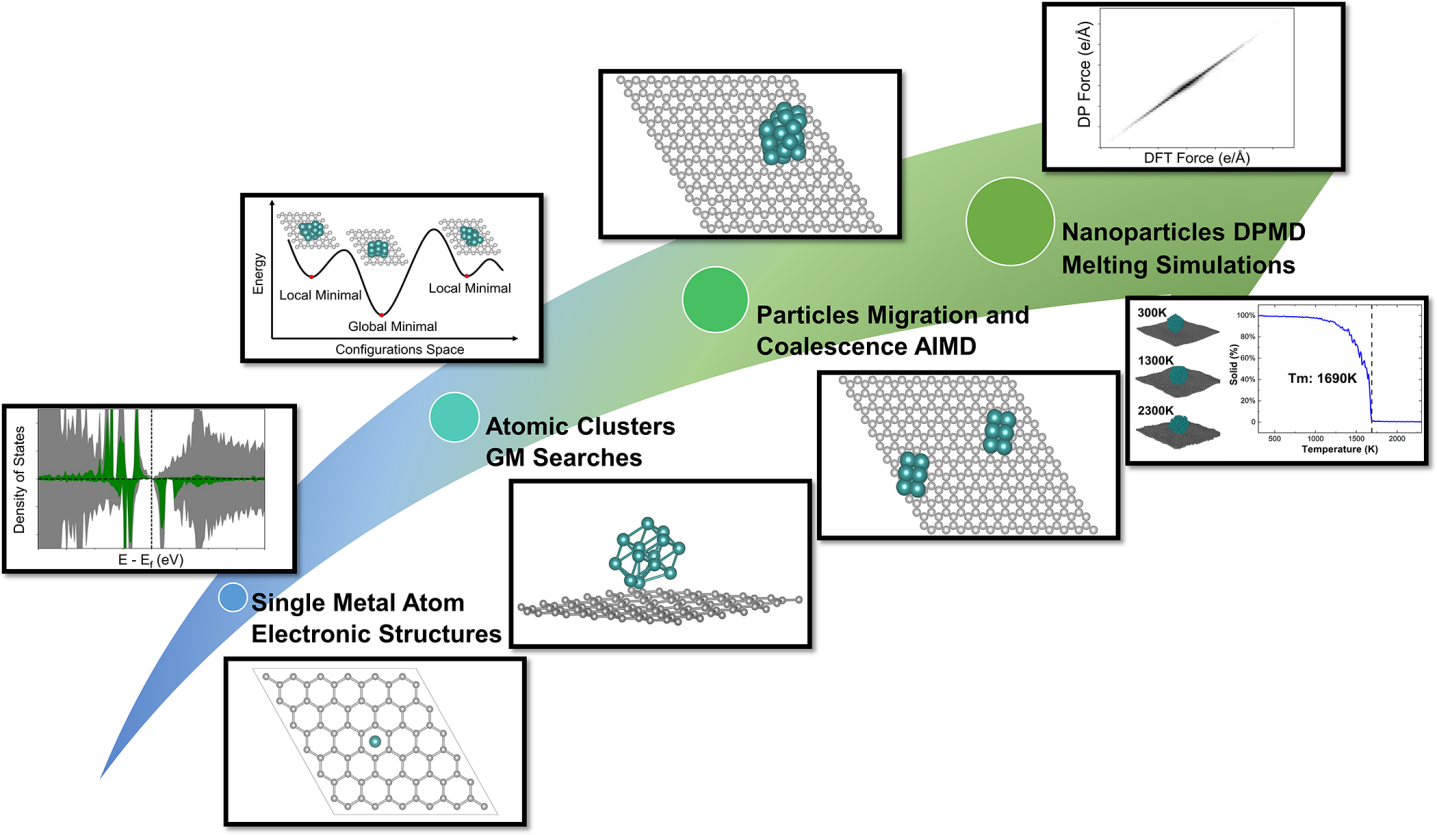
Schematic illustration of multiscale modeling. Model sizes increase from the bottom left to the top right. The multiscale modeling starts with electronic structure analysis of supported Ru SMAs, followed by a GM search of supported Ru atomic clusters. The aggregation of GM atomic clusters was then simulated by AIMD. The data gained in first-principles calculations were collected for DPMD force field construction and studying the melting of Ru nanoparticles.

Extensive research has demonstrated that N-doped graphene comprises graphitic, pyridinic, and pyrrolic N-doping sites, as compared to undoped regions.^13,14^ While graphitic N-doped graphene exhibits electronic n-type doping (Fermi level shifting to a higher energy value), pyridinic and pyrrolic N-doped graphene display p-type characteristics (Fermi level shifting to a lower energy value) in comparison to pristine graphene.^15,16^ We have constructed three representative models of pristine graphene (PG), graphitic N-doped graphene (N1), and pyridinic N-doped graphene (N1V1) as potential carbon supports for comparison, on the basis of experimental observations (top panel in Figure 2a).^17−19^ Our projected density of states (PDOS) calculations for the three carbon supports (Figure 2b, top panel) indicate that N1 and N1V1 exhibit ∼0.3 eV n-type and p-type Fermi level shifts, respectively, which is consistent with previous studies.^15,16^

**Figure 2:**
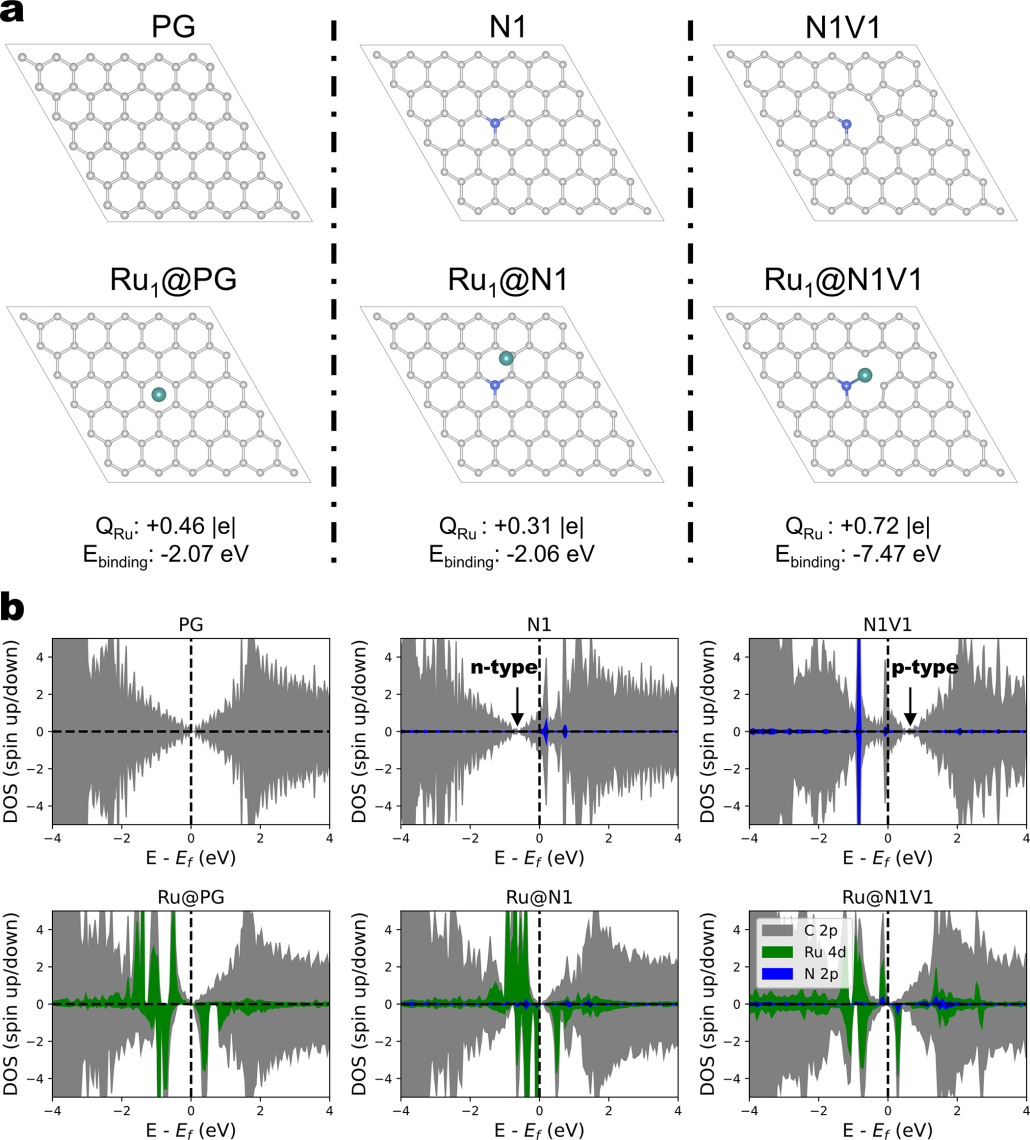
(a) Optimized structures of bare supports (top panel) and Ru SMA adsorbed on 6 × 6 pristine graphene (PG), graphitic N-doped graphene (N1), and pyridinic N-doped graphene (N1V1) (bottom panel). Carbon, nitrogen, and ruthenium atoms are colored gray, blue, and green, respectively. The Ru Bader charge (*Q*_Ru_) and Ru SMA binding energy (*E*_binding_) are listed below for each support. (b) Projected density of states (PDOS) of bare supports (top panel) and Ru SMA adsorbed on PG, N1, and N1V1. C 2p, N 2p, and Ru 4d refer to 2p orbitals of C and N, and 4d orbitals of Ru, respectively.

We subsequently introduced a Ru SMA onto the supports. The optimized structures displayed in Figure 2a (bottom panel) reveal that the Ru SMA prefers to adsorb on the hollow site of PG,^20^ a repulsion occurs between the graphitic N atom and Ru SMA, and the vacancy of N1V1 serves as an anchoring site for the Ru SMA. This variation in MSI is further supported by their binding energies (*E*_binding_) of −2.11, −1.93, and −7.28 eV for Ru SMA on PG, N1, and N1V1, respectively, as presented in Figure 2a. *E*_binding_ was calculated by
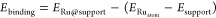
where *E*_Ru@support_ refers to the total energy of Ru SMA supported on substrates, *E*_Ru_atom__ refers to the energy of Ru SMA in vacuum, and *E*_support_ refers to the energy of the substrates. The decrease in MSI from N1V1 and PG to N1 can be attributed to the reduced level of charge transfer between the metal and support, as demonstrated by the Bader charge analysis in Figure 2a, where Ru SMAs donate 0.46, 0.31, and 0.72 |e| of electron to the supports. The relatively strong MSI between the Ru SMA and N1V1 can be explained by the carbon vacancy-induced p-type doping, which withdraws more electrons from the Ru. As a result, the most positive Bader charge of Ru in Ru/N1V1 is observed. In contrast, the n-type doping of N1 inhibits the withdrawal of an electron from Ru, leading to a weaker MSI compared to that of the pristine graphene support. In summary, the loss of an electron from the Ru SMA occurred when it interacts with the support. Nevertheless, the electron affinity of the supports can be controlled by different doping sites, resulting in a variety of MSI.

In addition to SMAs, recent studies have frequently reported carbon-supported atomic clusters, such as N-doped graphene-supported Ru for advanced catalysis.^4,21^ To accurately characterize the configurations of carbon-supported Ru atomic clusters, we conducted DFT GM searches using an evolutionary algorithm. The GM atomic cluster configurations of Ru_2–13_ are presented in Figure 3a. Although 13 represents the minimal magic number for icosahedral symmetry,^22^ previous GM searches have identified a nonsymmetric Ru_13_ cluster configuration in the gas phase,^23−25^ which also appears as the GM Ru_13_ structure on N1 in this study. Interestingly, we predicted a new Ru_13_ cluster structure showing a Ru atom slightly protruded toward the hollow site of PG and carbon vacancy of N1V1 supports, respectively.

**Figure 3:**
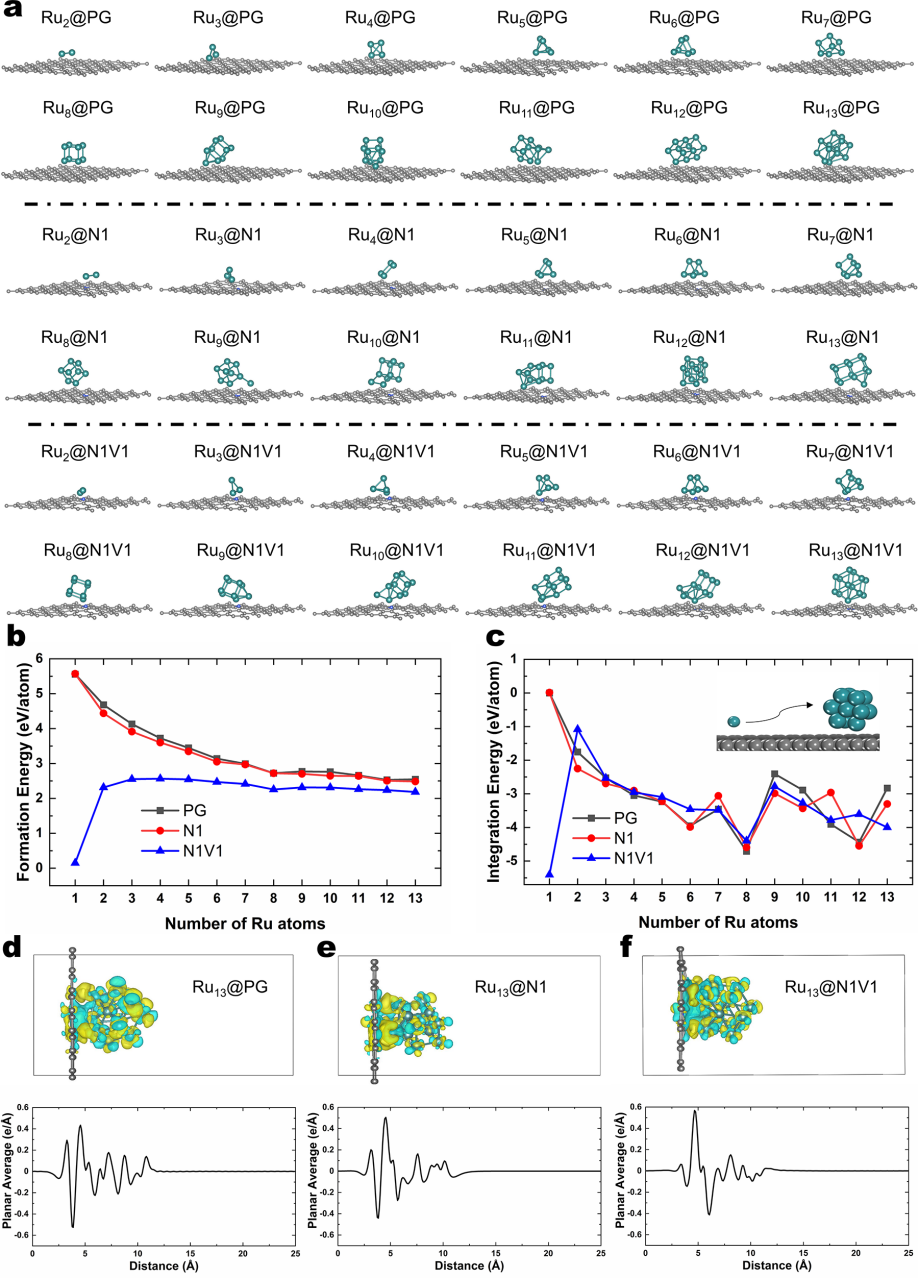
(a) GM Ru_2–13_ clusters supported on PG, N1, and N1V1. (b) and (c) Formation and integration energies of GM Ru_1–13_ clusters supported on PG, N1, and N1V1. (d)–(f) Charge density differences of GM Ru_13_ clusters supported on PG, N1, and N1V1, respectively (yellow and blue isosurfaces indicate increases and decreases in electron density by 0.002 |e|/Å^3^, respectively). Corresponding integration of the charge density difference along the support plane is provided below.

To quantitatively analyze the GM atomic clusters configurations, we employed the formation energy (*E*_formation_) as defined below:
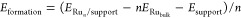
where *E*_Ru_*n*_/support_ represents the energy of a supported SMA or a Ru cluster (*n* = 2–13), *E*_Ru_bulk__ denotes the energy of bulk Ru per atom, and *E*_support_ refers to the energy of the individual support (PG, N1, or N1V1). The decrease in formation energy is shown in Figure 3b as the number of Ru atoms increases, indicating a trend of aggregation of Ru atomic clusters on PG and N1. In addition, although N1V1 can stabilize Ru SMA, the energy of formation of the Ru cluster is lower than those with other supports, which might attract Ru to aggregate on it. Given the strong MSI between the Ru SMA and N1V1 support, a low formation energy value is observed. To give a fair comparison of the stability between different sizes of Ru atomic clusters, we also employed the integration energy as defined below:
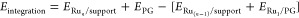
where *E*_PG_ refers to the energy of the PG support and *E*_Ru_(*n*–1)_/support_ refers to the energy of a supported Ru_(*n*–1)_ cluster (when *n* = 1, the integration energy refers to the energy of the Ru SMA diffusing from PGN1 or N1 V1). The integration energy shown in Figure 3c could be utilized to identify how easily the Ru SMA on a nearby graphene support^26−28^ migrates to the metal cluster on a specific support. This methodology provides another approach for comparing the stability between various sizes of Ru atomic clusters on different supports. Among Ru_2–13_-supported clusters, the integration energy analysis reveals that Ru_8_ (simple cubic) on all supports is more stable than the other atomic clusters.

For deeper insight into the electronic structures of supported Ru atomic clusters, we conducted charge density difference analysis (Figure 3d–f) for Ru_13_ clusters on the PG, N1, and N1V1 supports. For PG and N1 supports, the integration of the charge density difference suggests that the charge transfer from the support to the Ru cluster is strong at the interface (around 3.5–6 Å in Figure 3d–f, bottom panel). The Ru_13_ cluster seems to accept electrons from the support of PG or N1, leaving a negative peak (losing electrons) at the support and a positive peak (gaining electrons) around the Ru atoms. On the contrary, for the N1V1 support, the most negative and positive peaks appear on Ru atoms. Additionally, the negative peak on the N1V1 support is lower than those on PG and N1. These data imply that there might be charge redistribution on the metal cluster of the Ru_13_@N1V1, compared to the cases for PG and N1.

A GM search does not guarantee the stability of atomic clusters, especially at the working temperature of the catalysts (e.g., ∼700 K) due to particle sintering. According to the volcano curve of MSI and particle sintering process,^29^ strong MSI leads to a flat nanoparticle and leaves a large contact area between the particle and support, causing particles to sinter through the Ostwald ripening mechanism. Conversely, weak MSI results in a spherical nanoparticle and leaves a small contact area between the metal particle and support, leading to sintering through particle migration mechanisms.^30^ Ru and pristine graphene exhibit a typical weak MSI, as evidenced by the rather spherical GM Ru atomic clusters and the weak binding energy of the Ru SMA in the previous sections.

Thus, the sintering of Ru particles on graphene is expected to occur through the particle migration and coalescence mechanism, while N1V1 can serve as an anchor to suppress sintering due to their alternating MSI strength.^31^ To verify the anchoring effect, we construct a support with two N1V1 sites, followed by adsorption of two GM Ru_13_ atomic clusters to conduct 20 ps AIMD simulations (Figure 4a, bottom panel) at a temperature of 700 K. As a high concentration of graphitic N-doping sites has been reported,^32^ we also constructed a N1 support model with regions with a high concentration of graphitic N-doping sites (Figure 4a, middle panel) to see whether it can suppress particle coalescence as a barrier. As a reference, the AIMD of two Ru_13_ Atomic clusters supported on PG is also conducted, as shown in the top panel of Figure 4a.

**Figure 4:**
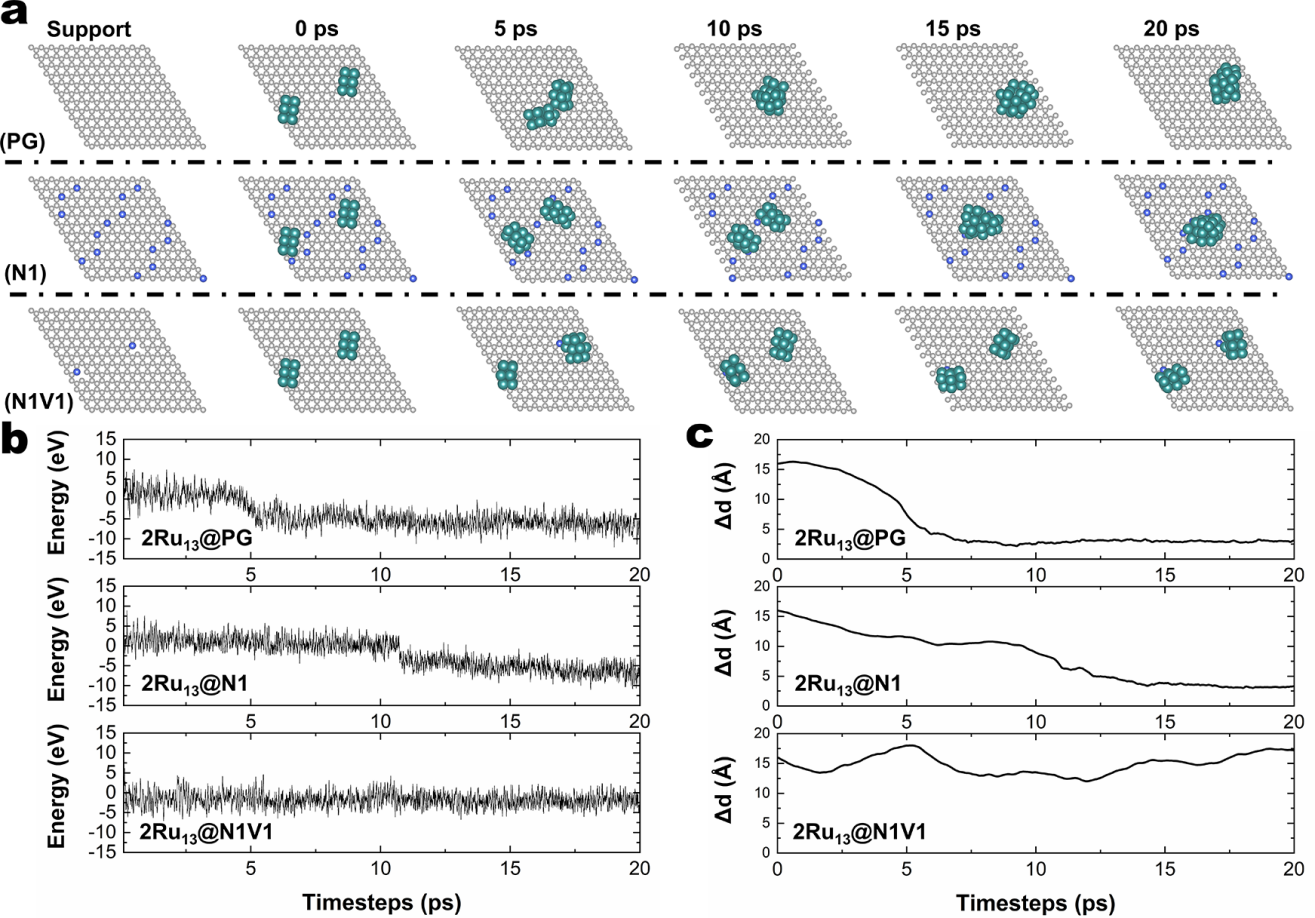
AIMD simulations of two GM Ru_13_ atomic clusters supported on PG, N1, and N1V1 at 700 K. (a) Bare support structures and snapshots of two supported Ru_13_ atomic clusters AIMD simulations at 0, 5, 10, 15, and 20 ps. (b) Potential energy fluctuations of two GM Ru_13_ clusters supported on PG, N1, and N1V1. (c) Mass center distances between the two GM Ru_13_ clusters (Δ*d*) during the AIMD simulations.

The snapshots of the AIMD simulations (Figure 4a), the potential energy fluctuations (Figure 4b), and the statistics of the distances between the mass centers of two clusters (Figure 4c) demonstrate that the strong MSI of pyridinic N-doped support can suppress particle coalescence, with the two Ru_13_ atomic clusters remaining separated throughout the 20 ps AIMD simulation. In contrast, the graphitic N-doped support slows the coalescence of two Ru_13_ clusters, compared to the pristine graphene support. However, the two Ru atomic clusters still aggregate in the end.

We have demonstrated that Ru anchoring on N1V1 sites can effectively preserve single atoms or atomic clusters against sintering, whereas N1 sites show less affinity for Ru than does PG. Therefore, to study the morphology of supported nanoscale Ru particles, we conducted a DPMD simulation using PG as the support. The thousands of atoms in nanoscale particles make DFT calculations infeasible, particularly when considering the support in this study. However, classical MD force fields might not accurately represent the MSI.^30^ On the basis of the AIMD data set, we trained a ML-based force field and utilized it to simulate the melting process of a series of Ru nanoparticles from 147 to approximately 20 000 atoms. Although Ru is generally classified as a HCP-crystallized noble metal, Ru FCC nanoparticles with a (111) facet have recently being synthesized.^33,34^ Therefore, we constructed nanoparticles of different sizes and shapes, including FCC icosahedron (Ih), truncated decahedron (Dh), and Wulff construction of a HCP single crystal (HCP), to further study the morphology of nanoscale Ru particles supported on PG.

The thermal stabilities of these Ru nanoparticles were determined by DPMD-simulated melting points (Figure 5), rather than relying on the well-studied surface excess energy,^35^ as the latter could be ambiguous when considering supports.^36^ Theoretical melting points were determined by calculating the percentage of solid atoms that change with temperature during DPMD simulation using bond-orientational order.^37,38^

**Figure 5:**
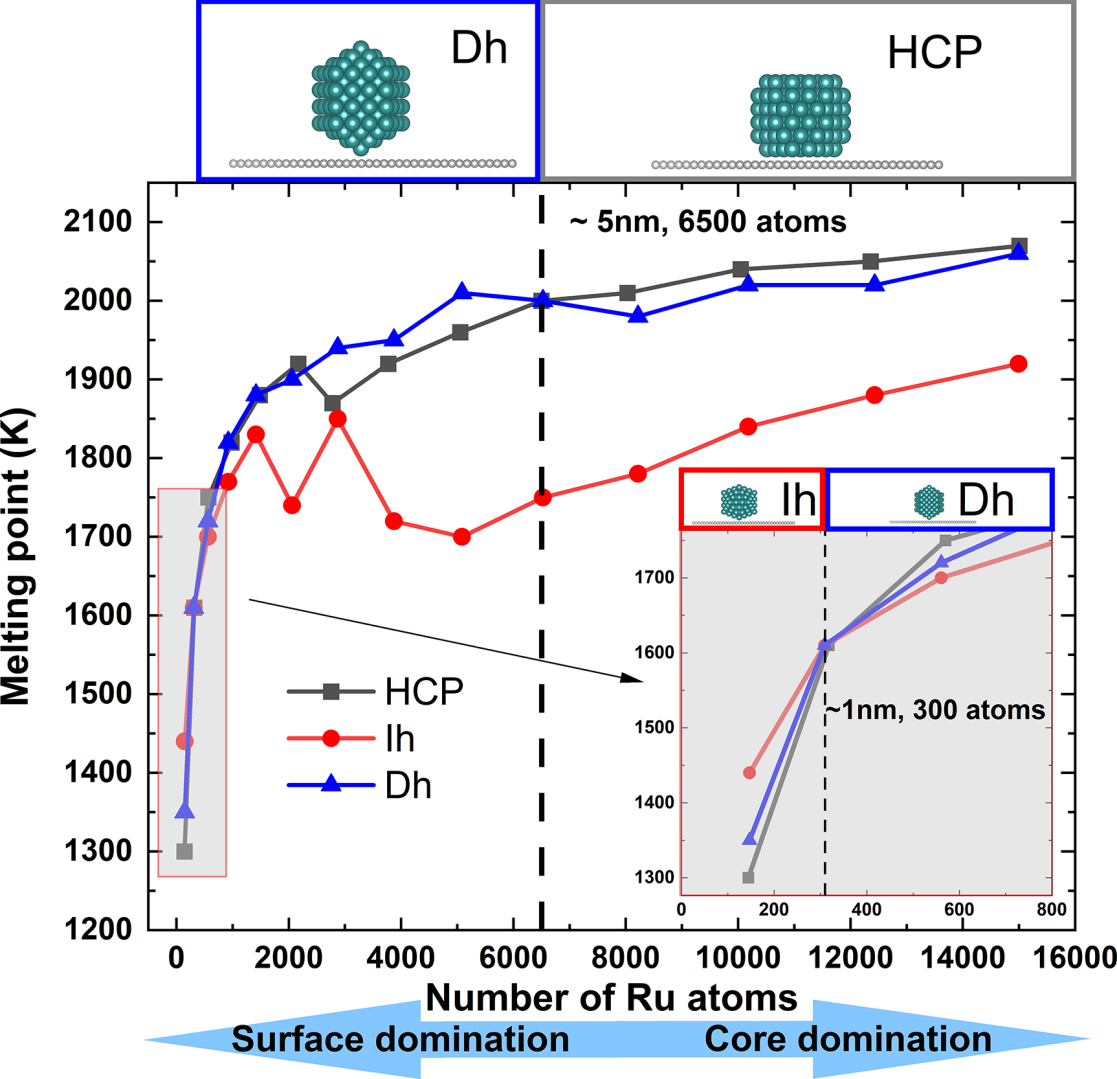
DPMD-simulated melting points of icosahedron (Ih), truncated decahedron (Dh), and hexagonal close packed Wulff construction (HCP) nanoparticles on PG with each configuration above the thermally stable regions. An enlarged view of 0–1000 Ru atoms (gray area) provides better insight into small nanoparticles. The crossover points of Ih to Dh (∼1 nm diameter, 300 atoms) and Dh to HCP (∼5 nm diameter, 6500 atoms) are shown with dashed reference lines. The correlation between surface atom domination and core atom domination for small and large nanoparticles is illustrated below.

The calculated melting point can be used to identify the stability for various morphologies of a specific dimension of nanoparticles. The inset of Figure 5 indicates that the thermally favored structure for the Ru nanoparticles within 1 nm is the Ih configuration. This is due to the Ih nanoparticle consisting of 20 individual tetrahedra, leading to a surface:volume ratio lower than that of HCP (Figure S1) and a shape more spherical than that of Dh (with fewer atoms at edge and corner sites).^34^ However, the abundance of a grain boundary inside the Ih configuration, as shown in Figure S2, is greater than that of the Dh configuration.^39^ Consequently, Dh becomes the dominant structure for Ru nanoparticles from 1 to 5 nm. Beyond 5 nm, the significance of decreasing the surface:volume ratio decreases, and the single-crystal HCP nanoparticles are dominant.

This trend is consistent with the findings for unsupported Ru nanoparticles^40^ and SiO_2_-supported Ag nanoparticles.^41,42^ However, because Ru has a HCP-crystallized structure, the stability turning size predicted in this study is lower than that for other noble metals. The shift in the thermally favored structure that changes with an increase in particle size can be attributed to the domination of the surface atoms gradually being replaced by that of the core as demonstrated at the bottom of Figure 5.

More precisely, the formation of nanoparticles follows the principle of the energy minimum, which leads to two different mechanisms of nanoparticle growth for surface atoms and core atoms. For surface atoms, because they have lower coordination numbers, they are in higher-energy states compared with the core atoms. To minimize the total energy of the nanoparticle, a minimum amount of surface atoms will be preferred. Thus, attempting to keep the spherical shape is a way to minimize the amount of surface atoms and reduce the total energy. With respect to the core atoms, because the bulk Ru metal is in the HCP structure, the more grain boundary is inside the core, the higher the energy is. However, those two mechanisms conflict with each other. In general, the more faces of a polyhedron (exhibiting more grain boundaries inside), the closer to a spherical shape. A spherical nanoparticle would leave many grain boundaries inside, while a single-crystal HCP core would lead to a sharp edge at the surface.^39,43^ Because the percentage of surface atom decreases with an increase in particle size, a small nanoparticle would prefer a spherical structure (e.g., icosahedron), while a single-crystal structure (Wulff construction for an HCP crystal) begins to dominate at a large scale. Such a conflict between surface and core domination in a certain range (1–5 nm in Figure 5) may result in an abundance of defect sites (such as B5 active sites), thus improving the catalytic performance and behavior of Ru nanoparticles of that size.^1,2,5,44^ This finding can be utilized to explain how the 1–5 nm Ru nanoparticles supported on the carbon materials give high activity in ammonia synthesis under mild conditions.

In conclusion, we developed a multiscale simulation strategy, including electronic structures, a GM search, AIMD, and DPMD, to examine the morphology and MSI of Ru supported on both pristine and N-doped graphene. Our electronic structure calculations unveiled strong MSI of SMA at pyridinic N-doping sites, owing to p-type electronic doping states originating from carbon vacancy. The GM search uncovered a series of Ru atomic clusters at various N-doped sites, and among them, simple cubic Ru_8_ clusters demonstrate the highest stability. The AIMD simulations suggested an anchoring effect at pyridinic N-doping sites, effectively inhibiting particle coalescence. In addition to DPMD, we simulated the melting behavior of nanoscale Ru particles in icosahedral (Ih), truncated decahedral (Dh), and HCP single-crystal configurations, identifying two thermal stability turning points at approximately 1 and 5 nm. These shifts can be attributed to the stronger influence of core atoms over the surface providing vital clues to their catalytic behavior. Our multiscale modeling strategy offers a detailed perspective on carbon-supported Ru, providing valuable insights for both theorists and experimentalists. More importantly, this multiscale modeling framework connected calculations in various scales and can be readily extended to other supported metal nanoparticle systems, thereby highlighting its broader implications for the field of nanotechnology.

## Supporting Information Available

DPMD ML force field for Ru on N-doped graphene (ZIP)
Surface atom percentage; grain boundary of Ih, Dh, and HCP nanoparticles; first principles calculations; convergence of the atomic GM search; RMSE of the energy and force between the DFT calculations and ML force field; benchmark study of the ML force field; nanoparticle DPMD melting simulations (PDF)
