# Supplementary material for: Unveiling the Morphology of Carbon-Supported Ru Nanoparticles by Multiscale Modeling
Source: Nano Lett. 2024 Jan 29;24(9):2689–97. doi: 10.1021/acs.nanolett.3c03796 (PMC10921456; doi:10.1021/acs.nanolett.3c03796)
Supplement: Supplementary file 2 — nl3c03796_si_002.pdf [file nl3c03796_si_002.pdf]

SUPPORTING INFORMATION

Unveiling the Morphology of Carbon Supported Ru Nanoparticles by Multiscale Modeling

Wenye Xuan[1, 2], Yu-Hao Liu[1], Shih-Yuan Chen[3], Matthew S. Dyer[2, 4], Hsin-Yi Tiffany Chen[1, 5, 6*]

[1]*Department of Engineering and System Science, National Tsing Hua University, Hsinchu 300044, Taiwan*

[2]*School of Chemistry, University of Liverpool, Crown St, Liverpool, L69 7ZD, United Kingdom*

[3]*Energy Catalyst Technology Group, Energy Process Research Institute (EPRI), National Institute of Advanced Industrial Science and Technology (AIST), 16-1 Onogawa, Tsukuba, Ibaraki 305-8569, Japan*

[4]*Materials Innovation Factory, University of Liverpool, 51 Oxford St, Liverpool, L7 3NY, United Kingdom*

[5]*College of Semiconductor Research, National Tsing Hua University, Hsinchu 300044, Taiwan*

[6]*Department of Materials Science and Engineering, National Tsing Hua University, Hsinchu 300044, Taiwan*

Surface Atom Percentage of Icosahedron, Decahedron and HCP Wulff-construction Nanoparticles

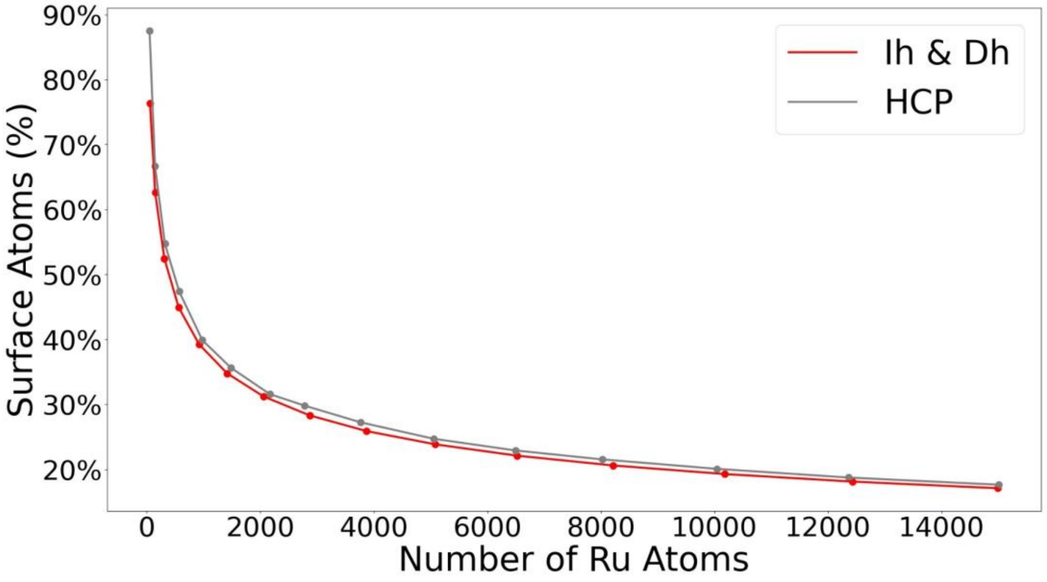

Figure S1. Surface atoms (outermost layer) percentage of Icosahedron (Ih), Decahedron (Dh) and HCP Wulff-construction nanoparticles with the increasing number of atoms.

Grain Boundary of Icosahedron, Decahedron and HCP Wulff-construction Nanoparticles

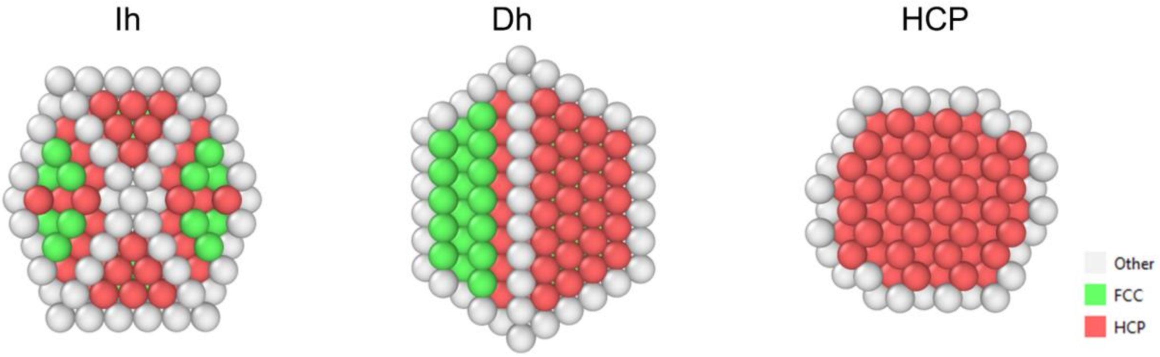

Figure S2. Cutaway views of the three Ru nanoparticles with atoms coloured by common neighbour analysis. FCC, HCP and non-crystalized atoms are coloured by green, red and grey respectively.

First Principles Calculations

First principles calculations were performed using the Vienna Ab initio Simulation Package (VASP)[1] with the projected augmented wave (PAW) potentials.[2] The exchange-correlation energy functional was described using the Perdew–Burke–Ernzerhof (PBE) generalized gradient approximation (GGA) with spin polarization.[3] To balance accuracy and computation speed, the parameters of DFT calculation were set differently for SMA DOS, atomic clusters GM search and AIMD as given in Table S1. The supports for SMA electronic structure calculations and atomic cluster GM search were constructed from a 6×6 pristine graphene (PG), graphitic N-doped graphene (N1), and pyridinic N-doped graphene (N1V1). As for AIMD, the support was extended to 12×12.

Table S1 Settings for density of states (DOS), global minimal (GM) search and ab-into molecular dynamics (AIMD) calculations.

Setting	DOS of SMA/Support	Atomic Clusters/Support Energy Alignment*	Atomic Clusters GM Search and AIMD
**Valence Electron**	Ru: $4p^6\ 4d^7\ 5s^1$<br>C: $2s^2\ 2p^2$	Ru: $4p^6\ 4d^7\ 5s^1$<br>C: $2s^2\ 2p^2$	Ru: $4d^7\ 5s^1$<br>C: $2s^2\ 2p^2$
**Cutting Off Energy**	450 eV	400 eV	300 eV
**Gaussian Smearing Width**	0.02	0.05	0.2
**Energy Convergence Criteria**	10^{-7} eV	10^{-5} eV	10^{-4} eV
**Force Convergence Criteria**	—	0.02 eV/Å	0.05 eV/Å
**K Points Mesh**	6x6x1	2x2x1	1x1x1
**DFT-D3 with BJ Damping**	**	**	**
**Time Step**	—	—	1 fs

* The GM atomic cluster structures were further optimized in a higher accuracy setting for energy alignment.

** We compared the calculation of binding energy of Ru SMA and formation energy of Ru atomic clusters with and without DFT-D3 correction. It seems that the difference of absolute energy value is limited, and the trend is consistent (Figure S3). To save computational resources we didn't include DFT-D3 correction in our calculations. As for AIMD and DPMD, we tried to add the DFT-D3 correction to the AIMD dataset using transfer-learning for the ML force field training.[4] However, the modified force field did not give a reasonable description to the nanoparticle melting behavior. Hence, the DFT-D3 correction was not included in the AIMD and ML force field. To keep the dataset consistent, other DFT calculations were not included in the training of ML force field.

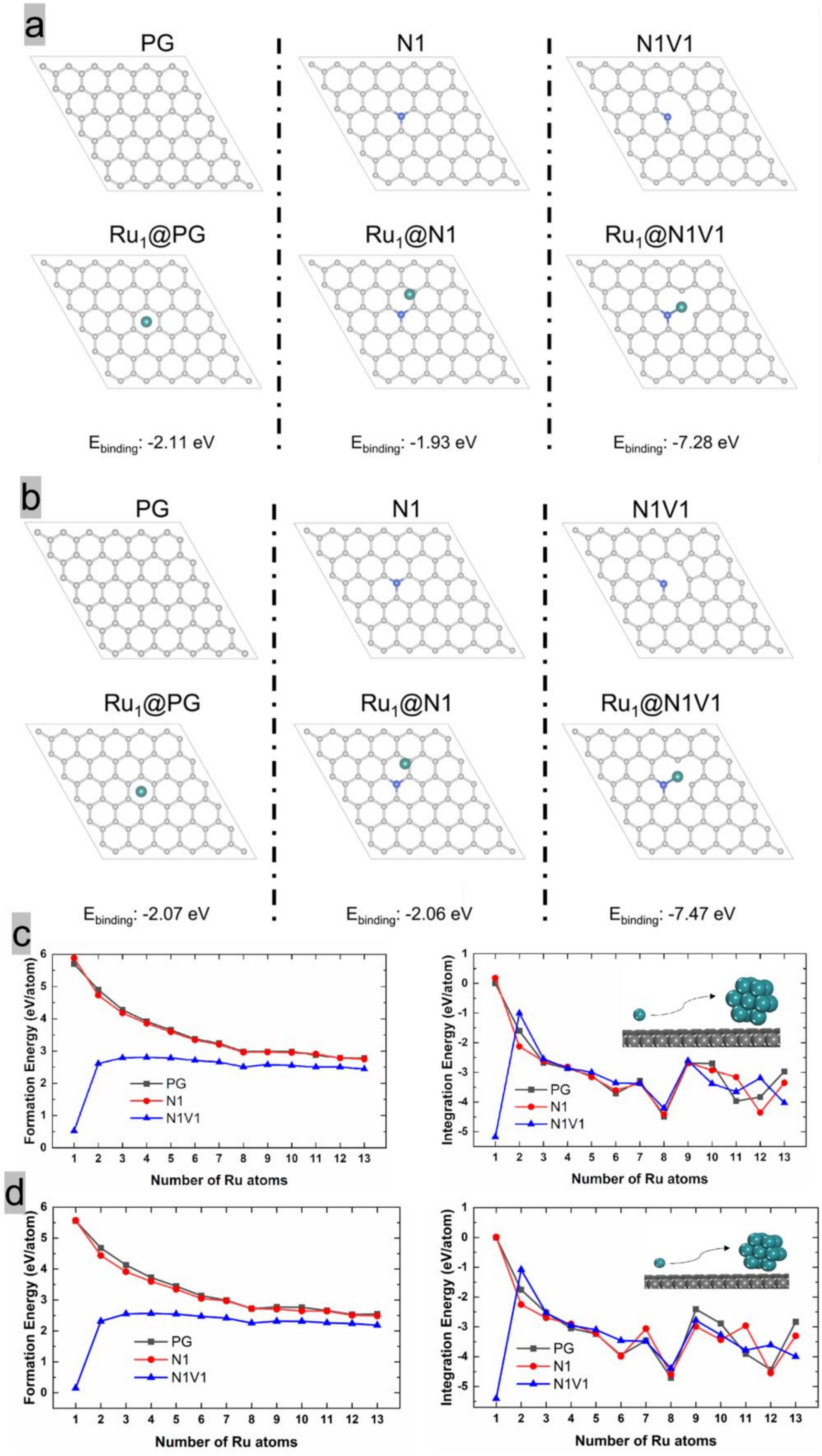

Figure S3, Binding energies of Ru SMA (a) without DFT-D3 correction and (b) with DFT-D3 correction. Formation energies and integration energies of Ru atomic cluster versus the number of Ru atoms on three supports (PG, N1 and N1V1) (c) without DFT-D3 correction and (d) with DFT-D3 correction.

Global Minimum (GM) Search

The Ru atomic clusters GM search was conducted using genetic algorithms implemented in the atomic simulation environment (ASE).[5, 6] The GM search for Ru_{2-13} on PG, N1, and N1V1 started with 20 initial candidates generated by randomly placing Ru atoms in a box with a quarter of the support area and a 5Å height above the doping site. After that, around 600 candidates for each atomic cluster were generated by crossing over and mutation (30% probability) from parent candidates. All candidates were optimized into local minimal before being used to generate new structures. The convergence of the GM search is given in Figure S4.

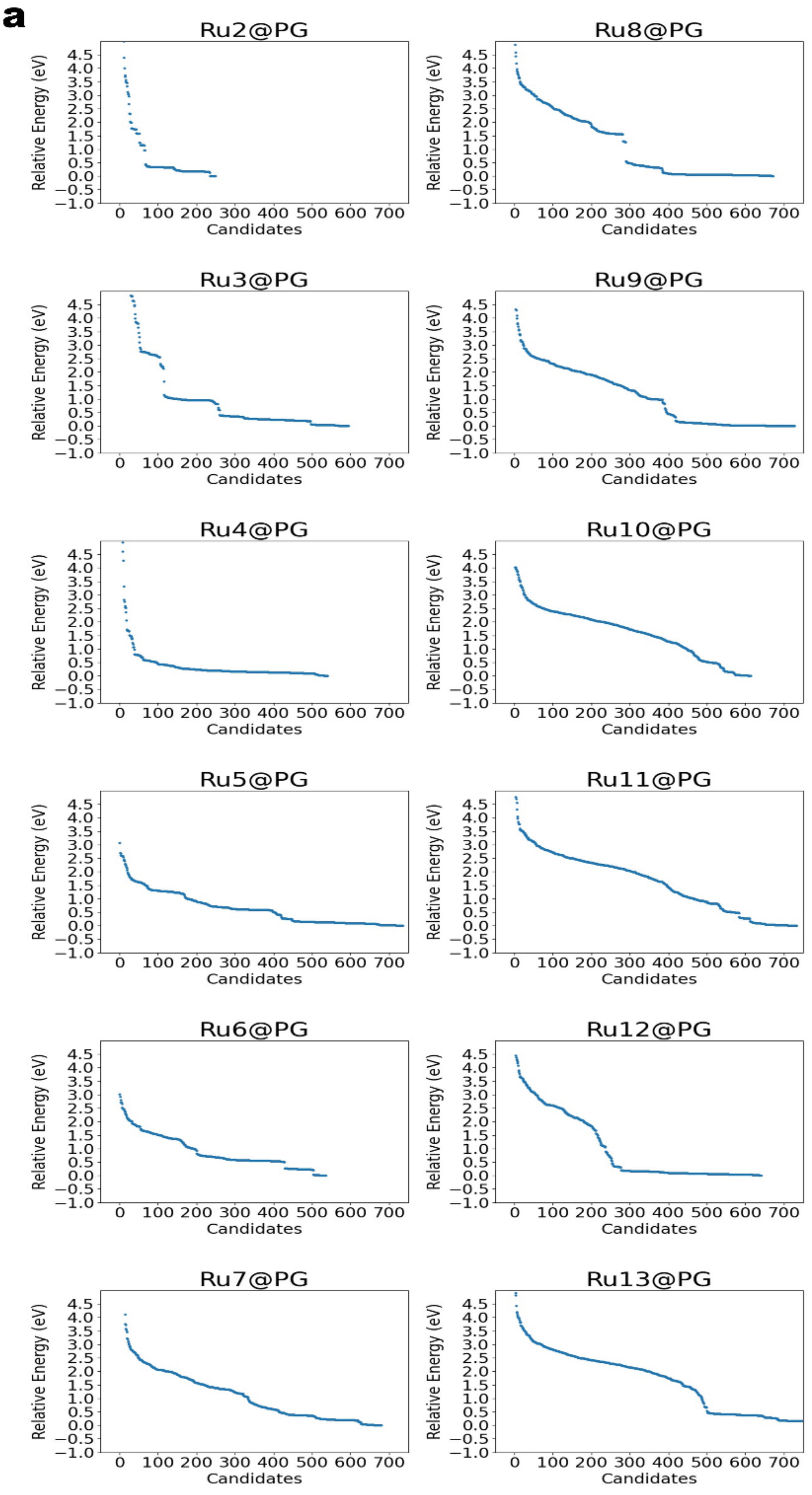
a
Ru2@PG
Ru8@PG
Ru3@PG
Ru9@PG
Ru4@PG
Ru10@PG
Ru5@PG
Ru11@PG
Ru6@PG
Ru12@PG
Ru7@PG
Ru13@PG
Relative Energy (eV)
Candidates

b

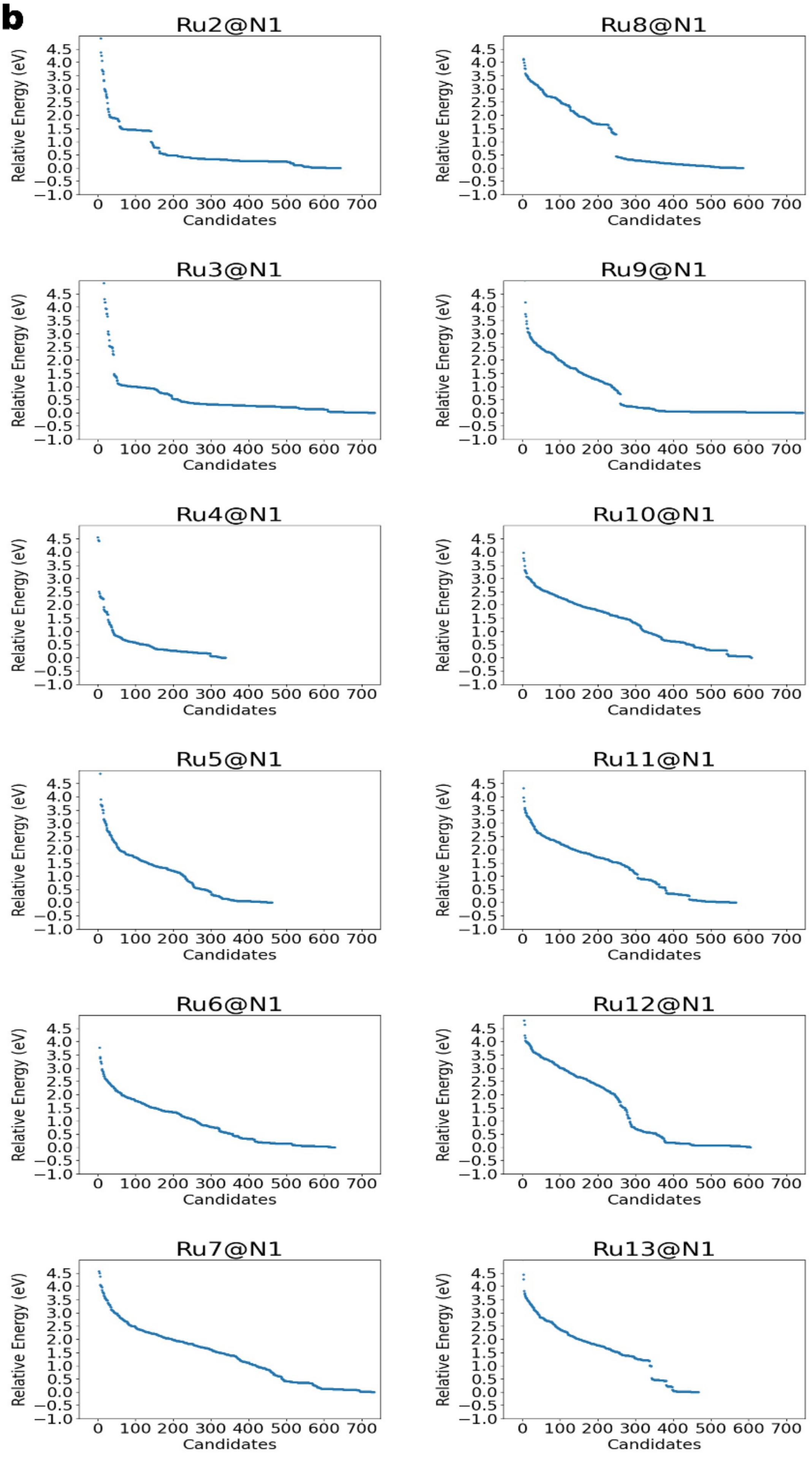
Ru2@N1
Ru8@N1
Ru3@N1
Ru9@N1
Ru4@N1
Ru10@N1
Ru5@N1
Ru11@N1
Ru6@N1
Ru12@N1
Ru7@N1
Ru13@N1
Relative Energy (eV)
Candidates

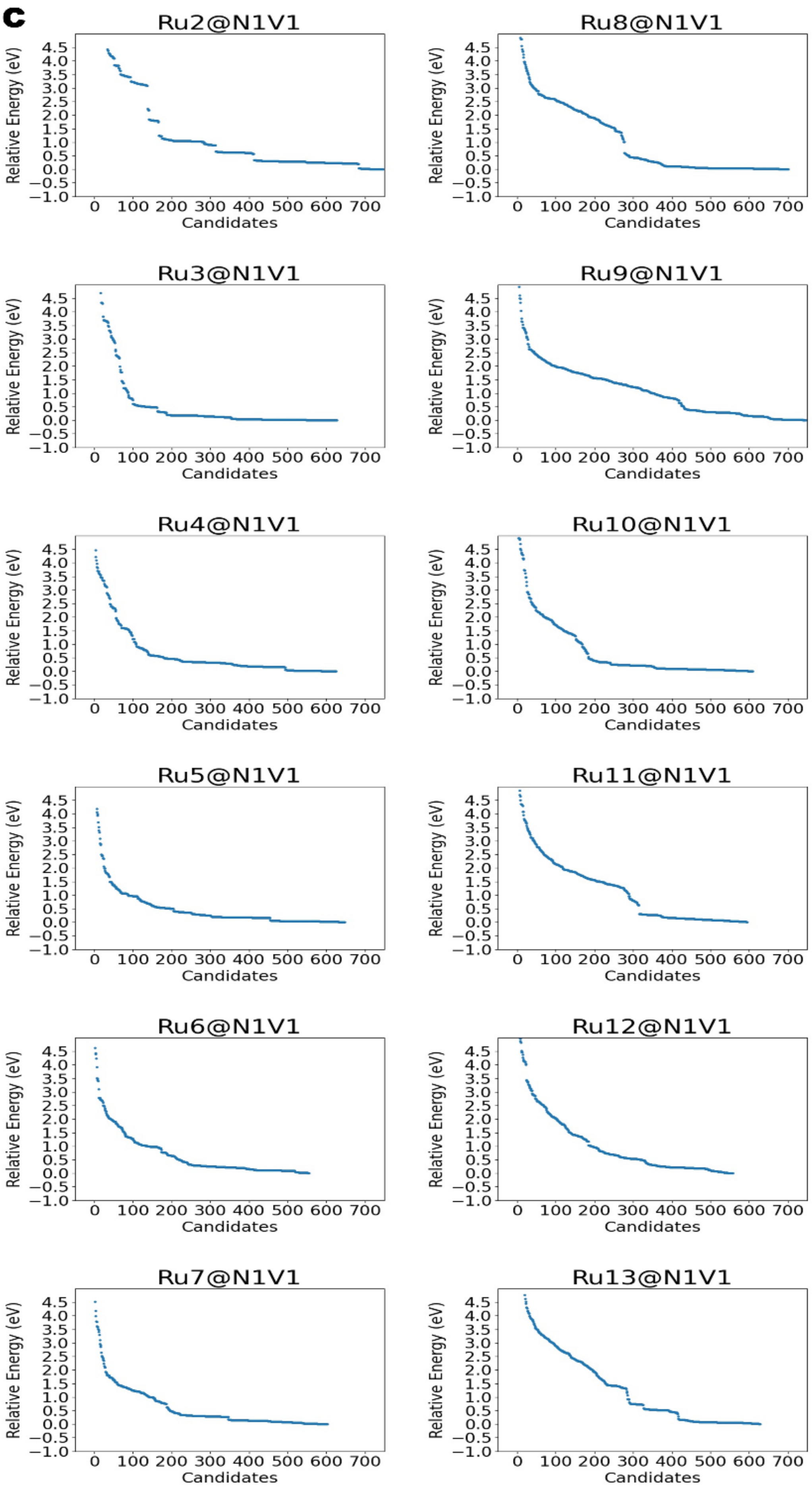

Figure S4. The energy convergence of the atomic clusters global minimal search on (a) PG, (b) N1 and (c) N1V1.

ML-based Force Field Training

The ML-based force field was trained using the DeepMD-kit package.[7] The embedding network was constructed by 3 layers each with (25, 50, 100) neurons after 5 million training iterations with a 7.0 Å cutting-off radius. To have a better description of wider configuration space, the training set also contains first principles calculation data obtained by active learning.[8, 9] The active learning was conducted using enhance sampling with the metadynamics method implemented in the PLUMED package,[10] with the Ru-Ru coordination number as the constrained value. The accuracy of the ML-based force field was further verified by the Root-mean-square error (RMSE) of supported Ih Ru_{55}, Dh Ru_{55} and HCP Ru_{48} nanoparticles melting process (300K – 2300K). As shown in Figure S5, the RMSE of energies and forces for Ih, Dh, and HCP nanoparticle melting are provided.

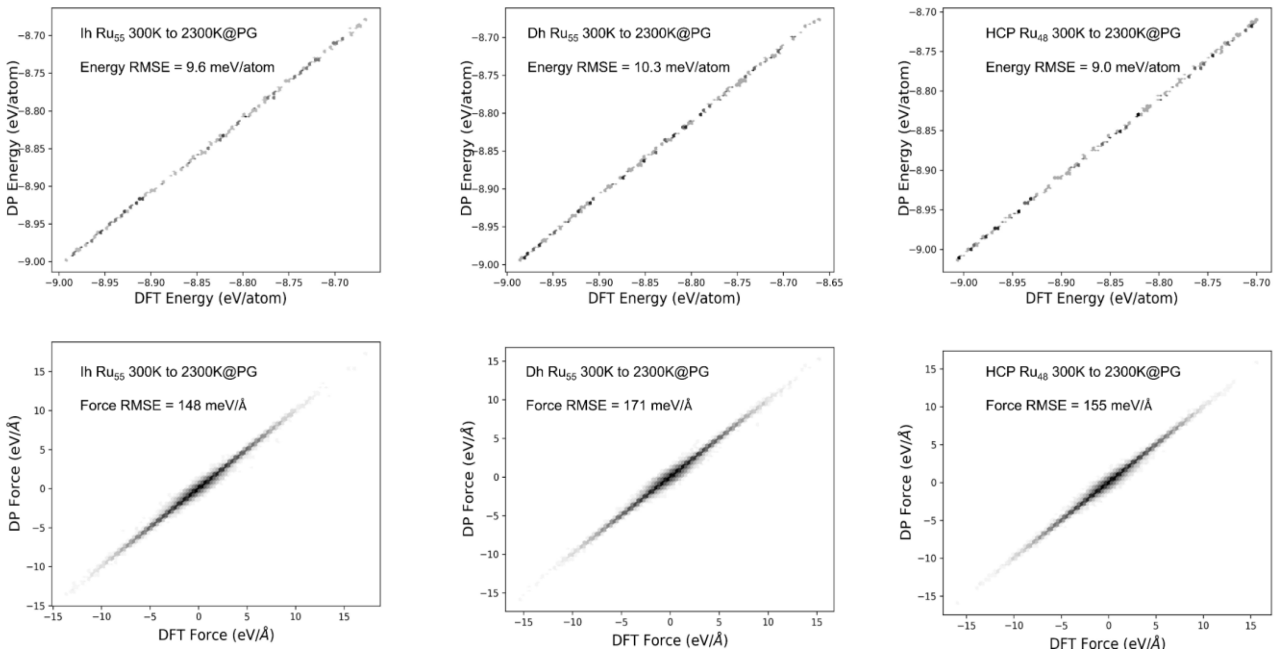

Figure S5. Root mean square error (RMSE) of the energy and force between DPMD force field and DFT result for PG supported Ih, Dh and HCP nanoparticle melting process.

Benchmark Study of the ML Force Field

A benchmark study between our ML force field and other established force field compared with DFT calculations for metal surface is given in Table S2. It should be noticed that a small number of energy RMSE does not promise the accuracy of ML force field. If the training set do not contain wider enough configuration space, the ML force field might have a poor performance in the testing set. As mentioned above, our training set contain a wide range of Ru nanoparticle configurations at different temperatures and the accuracy and universality could be guaranteed.

Table S2 The benchmark studies of ML force field for surface reactions

SYSTEM	Energy RMSE
Al (100) surface[11]	80 meV/atom
Pd/MgO[12]	~50 meV/atom
CO/Cu nanoparticle[13]	123meV/atom
Au/SiO_2 and Au/CeO_2[14]	~2 meV/atom
Au_{20}[15]	~4 meV/atom
Ag-Au alloy surface reconstruction[4]	6 meV/atom
This Study	9 meV/atom

Nanoparticle DPMD Melting Simulations

A series of Ru nanoparticles were constructed by the ASE nanoparticle module and Wulffpack.[16] The nanoparticles were than introduced into a simulation box (***a*** = ***b*** = 12 nm and ***c*** = 10 nm; $\boldsymbol{\alpha} = \boldsymbol{\beta}$ = 90° and $\boldsymbol{\gamma}$ = 120°) covered by pristine graphene. Each supported nanoparticle system was relaxed by DPMD at 300K for 100 ps first. Subsequently, the melting process was simulated by increasing the temperature of the system from 300K to 2300K with a temperature gradient of 10K each having a timescale of 10 ps (1 fs per ionic step). The solid and liquid phase exchange was determined by the Steinhardt bond-order parameter[17]

$$\boldsymbol{q_l(i) = (\frac{4\pi}{2l+1}\sum_{m=-l}^{l}\left|q_{l_m}(i)\right|^2)^{\frac{1}{2}}}$$

where,

$$\boldsymbol{q_{l_m}(i) = (\frac{1}{N(i)}\sum_{j=1}^{N(i)} Y_{l_m}(r_{ij})}$$

in which $\boldsymbol{Y_{l_m}}$ are the spherical harmonics and $\boldsymbol{N(i)}$ is the number of neighbours of particle i, $\boldsymbol{r_{ij}}$ is the vector connecting particles i and j. l and m are both integers with m∈ [−l, +l].[18] The neighbour list was determined by a cutting off radius of 3 Å. For atoms i and j, their bond was regarded as solid bond if

$$\boldsymbol{S_{ij} = \sum_{m=-6}^{6} q_{6_m}(i) q_{6_m}(i)^* \geq 0.5}$$

where 0.5 is a threshold setting based on experimental result.[19] The solid atom percentage changing with increasing temperature for a series of particle sizes of the three types of nanoparticle are given in Figure S6.

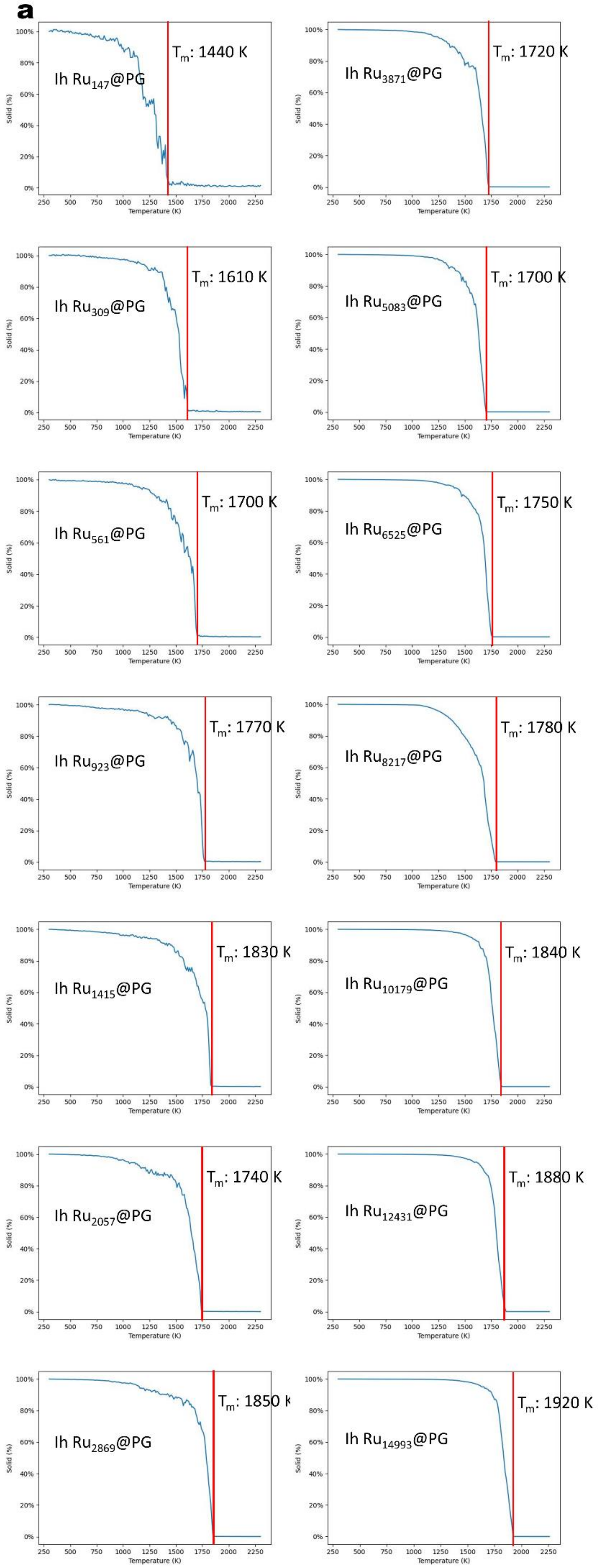
a
Tm: 1440 K
Ih Ru147@PG
Tm: 1720 K
Ih Ru3871@PG
Tm: 1610 K
Ih Ru309@PG
Tm: 1700 K
Ih Ru5083@PG
Tm: 1700 K
Ih Ru561@PG
Tm: 1750 K
Ih Ru6525@PG
Tm: 1770 K
Ih Ru923@PG
Tm: 1780 K
Ih Ru8217@PG
Tm: 1830 K
Ih Ru1415@PG
Tm: 1840 K
Ih Ru10179@PG
Tm: 1740 K
Ih Ru2057@PG
Tm: 1880 K
Ih Ru12431@PG
Tm: 1850 K
Ih Ru2869@PG
Tm: 1920 K
Ih Ru14993@PG
Solid (%)
Temperature (K)

b

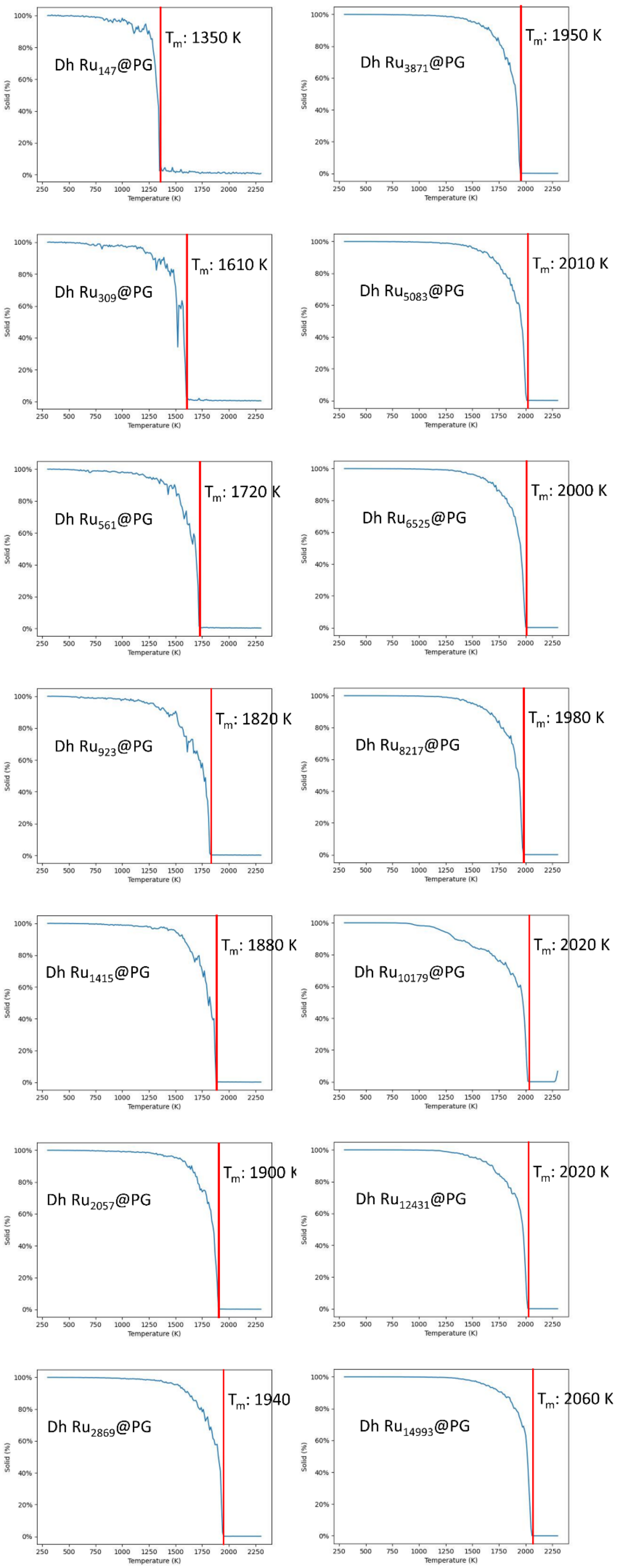
Dh Ru_{147}@PG
T_m: 1350 K
Dh Ru_{3871}@PG
T_m: 1950 K
Dh Ru_{309}@PG
T_m: 1610 K
Dh Ru_{5083}@PG
T_m: 2010 K
Dh Ru_{561}@PG
T_m: 1720 K
Dh Ru_{6525}@PG
T_m: 2000 K
Dh Ru_{923}@PG
T_m: 1820 K
Dh Ru_{8217}@PG
T_m: 1980 K
Dh Ru_{1415}@PG
T_m: 1880 K
Dh Ru_{10179}@PG
T_m: 2020 K
Dh Ru_{2057}@PG
T_m: 1900
Dh Ru_{12431}@PG
T_m: 2020 K
Dh Ru_{2869}@PG
T_m: 1940
Dh Ru_{14993}@PG
T_m: 2060 K
Solid (%)
Temperature (K)

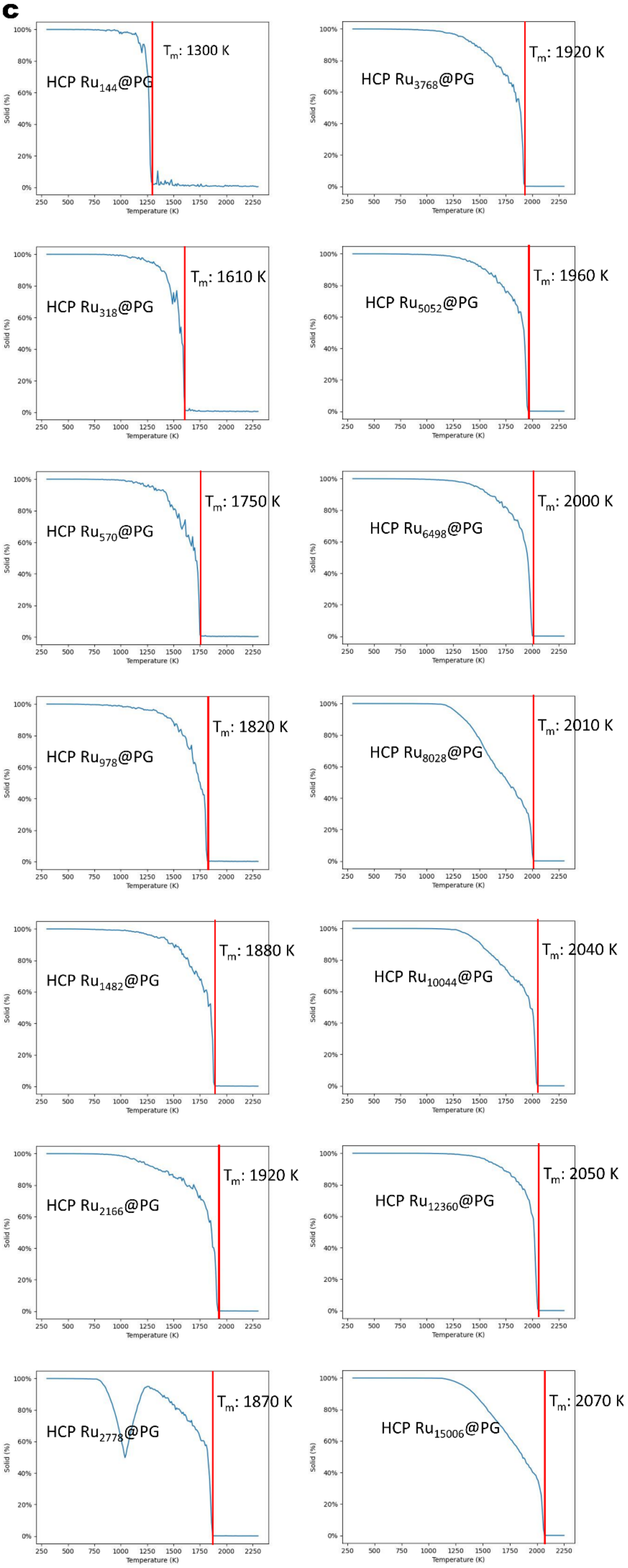

Figure S6. The solid atoms percentage of a series nanoparticles in Ih (a), Dh (b) and HCP (c) structures with the increasing temperature and their melting points (T_m , solids < 1%)

References

1. Kresse, G.; Hafner, J., ABINITIO MOLECULAR-DYNAMICS FOR LIQUID-METALS. *Phys. Rev. B* **1993,** *47* (1), 558-561.
2. Kresse, G.; Joubert, D., From ultrasoft pseudopotentials to the projector augmented-wave method. *Physical review b* **1999,** *59* (3), 1758.
3. Perdew, J. P.; Burke, K.; Ernzerhof, M., Generalized gradient approximation made simple. *Physical review letters* **1996,** *77* (18), 3865.
4. Wang, Y.; Zhang, L.; Xu, B.; Wang, X.; Wang, H., A generalizable machine learning potential of Ag–Au nanoalloys and its application to surface reconstruction, segregation and diffusion. *Modelling and Simulation in Materials Science and Engineering* **2021,** *30* (2), 025003.
5. Larsen, A. H.; Mortensen, J. J.; Blomqvist, J.; Castelli, I. E.; Christensen, R.; Dułak, M.; Friis, J.; Groves, M. N.; Hammer, B.; Hargus, C., The atomic simulation environment—a Python library for working with atoms. *Journal of Physics: Condensed Matter* **2017,** *29* (27), 273002.
6. Vilhelmsen, L. B.; Hammer, B., A genetic algorithm for first principles global structure optimization of supported nano structures. *The Journal of chemical physics* **2014,** *141* (4), 044711.
7. Wang, H.; Zhang, L.; Han, J.; Weinan, E., DeePMD-kit: A deep learning package for many-body potential energy representation and molecular dynamics. *Computer Physics Communications* **2018,** *228*, 178-184.
8. Zhang, Y.; Wang, H.; Chen, W.; Zeng, J.; Zhang, L.; Wang, H.; Weinan, E., DP-GEN: A concurrent learning platform for the generation of reliable deep learning based potential energy models. *Computer Physics Communications* **2020,** *253*, 107206.
9. Thompson, A. P.; Aktulga, H. M.; Berger, R.; Bolintineanu, D. S.; Brown, W. M.; Crozier, P. S.; in't Veld, P. J.; Kohlmeyer, A.; Moore, S. G.; Nguyen, T. D., LAMMPS-a flexible simulation tool for particle-based materials modeling at the atomic, meso, and continuum scales. *Computer Physics Communications* **2022,** *271*, 108171.
10. Promoting transparency and reproducibility in enhanced molecular simulations. *Nature methods* **2019,** *16* (8), 670-673.
11. Chapman, J.; Ramprasad, R., Nanoscale modeling of surface phenomena in aluminum using machine learning force fields. *The Journal of Physical Chemistry C* **2020,** *124* (40), 22127-22136.
12. Sauceda, H. E.; Gálvez-González, L. E.; Chmiela, S.; Paz-Borbón, L. O.; Müller, K.-R.; Tkatchenko, A., BIGDML—Towards accurate quantum machine learning force fields for materials. *Nature communications* **2022,** *13* (1), 3733.
13. Huang, Y.; Chen, Y.; Cheng, T.; Wang, L.-W.; Goddard III, W. A., Identification of the selective sites for electrochemical reduction of CO to C2+ products on copper nanoparticles by combining reactive force fields, density functional theory, and machine learning. *ACS Energy Letters* **2018,** *3* (12), 2983-2988.
14. Liu, J.-C.; Luo, L.; Xiao, H.; Zhu, J.; He, Y.; Li, J., Metal affinity of support dictates sintering of gold catalysts. *Journal of the American Chemical Society* **2022,** *144* (45), 20601-20609.
15. Fronzi, M.; Amos, R. D.; Kobayashi, R.; Matsumura, N.; Watanabe, K.; Morizawa, R. K., Evaluation of Machine Learning Interatomic Potentials for the Properties of Gold Nanoparticles. *Nanomaterials* **2022,** *12* (21), 3891.
16. Rahm, J. M.; Erhart, P., WulffPack: A Python package for Wulff constructions. *Journal of Open Source Software* **2020,** *5* (45), 1944.
17. Steinhardt, P. J.; Nelson, D. R.; Ronchetti, M., Bond-orientational order in liquids and glasses. *Physical Review B* **1983,** *28* (2), 784.
18. Menon, S.; Leines, G. D.; Rogal, J., pyscal: A python module for structural analysis of atomic environments. *Journal of Open Source Software* **2019,** *4* (43), 1824.
19. Auer, S.; Frenkel, D., Numerical simulation of crystal nucleation in colloids. *Advanced Computer Simulation: Approaches for Soft Matter Sciences I* **2005**, 149-208.
